# The double solid solution (Zr, Nb)_2_(Al, Sn)C MAX phase: a steric stability approach

**DOI:** 10.1038/s41598-018-31271-2

**Published:** 2018-08-24

**Authors:** Thomas Lapauw, Bensu Tunca, Daniel Potashnikov, Asaf Pesach, Offir Ozeri, Jozef Vleugels, Konstantina Lambrinou

**Affiliations:** 1 KU Leuven, Department of Materials Engineering, Kasteelpark Arenberg 44, B-3001 Leuven, Belgium; 2 SCK•CEN, Boeretang 200, B-2400 Mol, Belgium; 3 Israel Atomic Energy Commission, P.O. Box 7061, Tel-Aviv, 61070 Israel; 4 Physics Department, Nuclear Research Centre – Negev, Beer-Sheva, 84190 Israel; 5 Reactor Department, Nuclear Research Center-Soreq, Yavne, 81800 Israel

## Abstract

The addition of Nb and Sn to Zr_2_AlC is investigated, targeting the synthesis of a Zr-rich bulk MAX phase free of ZrC. The 211 phase formation in the two quaternary Zr-Nb-Al-C and Zr-Al-Sn-C systems is evaluated. Solubility over the entire compositional range in (Zr, Nb)_2_AlC and Zr_2_(Al, Sn)C is observed. In terms of effectiveness, the addition of Sn is preferred over the addition of Nb, as the former is selectively incorporated into the 211 structure. A combinatorial approach results in the formation of phase-pure (Zr_0.8_, Nb_0.2_)_2_(Al_0.5_, Sn_0.5_)C. The effect of the added solutes on the microstructure and crystallographic parameters is investigated. The addition of Nb and Sn reduces the distortion parameter of the trigonal prism compared to pure Zr_2_AlC. Therefore, an attempt is made to establish a more general stability criterion for the M_2_AC structure based on the steric relationship between the atoms in the M_6_A trigonal prism. Inspired by the Hume-Rothery rules, it is suggested that comparable atomic radii of the M- and A-atoms provide a good starting point to obtain a stable 211 MAX phase.

## Introduction

A group of layered ternary carbides and nitrides, commonly known as the MAX phases, is studied intensively in the last 20 years due to their remarkable set of properties^1,2^. The M_n+1_AX_n_ phases, where M is an early transition metal, A is an element from groups 12–15 in the periodic table, X is C and/or N and n is an integer commonly equal to 1, 2 or 3 (forming subgroups 211 – earlier referred to as the H-phases^2^, 312 and 413, respectively), combine the properties of ceramics with some merits of metals. These properties result from their laminated nature, as n M_6_X octahedra are alternated with atomic layers of A^3,4^.

Recently, the interest in Zr-based MAX phase carbides (Zr_n+1_AC_n_) arose, mainly triggered by their potential use in the nuclear sector. Zr is the preferred M-element as it has a small neutron cross-section for thermal and fast neutrons^5^, Al offers the potential of forming a protective Al_2_O_3_ layer in oxidative environments. C is preferred over N as X-element in order to avoid the production of the long-lived ^14^C radioisotope during irradiation. More specifically, Zr-based MAX phases are considered candidate coating materials for the protection of Zr-based alloy fuel clads designed to increase the accident tolerance of Gen-II/III LWRs (light water reactors)^6^. Alternatively, select MAX phases in bulk (monolithic) form are considered as structural materials for pump impellers intended for use in Gen-IV LFRs (lead-cooled fast reactors)^7^. This component is exposed to fast-flowing lead-based alloys, i.e., conditions in which conventional nuclear steel grades may suffer erosion/corrosion that leads to severe material loss^8^.

Although the ternary Zr_2_AlC phase could be synthesized, the material contained a significant amount of the undesirable ZrC phase, making the synthesis of phase-pure Zr_2_AlC MAX phase materials very challenging^9,10^. An alternative approach to obtain MAX phase pure material is to stabilize this stoichiometry by making (Zr, M)_2_AlC and Zr_2_(Al, A)C solid solutions^11^. Stable solid solutions on the M-site have been reported for M = Nb and Ti^12–14^, while recently the in-plane ordered (V_2/3_, Zr_1/3_)_2_AlC i-MAX phase was synthesized^15^. Horlait *et al*. successfully substituted Al with Sn, Pb, Sb and Bi^11,16^. However, ZrC or (Zr, M)C was reported as parasitic phase in all Zr_2_AlC-based solid solutions. In order to fully benefit from the aforementioned unique set of MAX phase properties, elimination of this high hardness, rock-salt-structured carbide phase is desired. Only when this is realised, the phase-pure material properties can be experimentally determined, allowing the exploitation of the full potential of these ternary ceramics for select applications in the nuclear field.

This work aims to synthesise a material, which contains only Zr_2_AlC-based MAX phases and is free from NaCl-structured carbides, like ZrC. Nb and Sn are selected as alloying elements on the M- and A-site, respectively, targeting a double solid solution with the (Zr_1−x_, Nb_x_)_2_(Al_1-y_ , Sn_y_)C general stoichiometry. The effect of these alloying elements on the crystal structure are evaluated.

## Methods

### Synthesis

**Figure 1:**
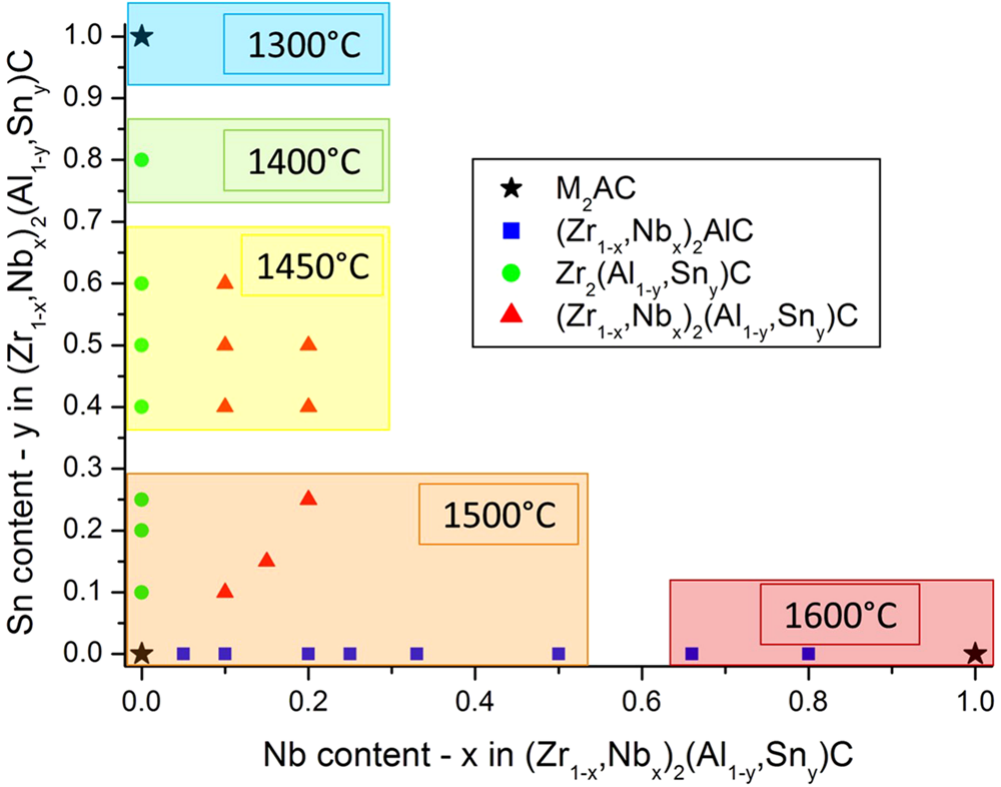
The various (Zr_1-x_, Nb_x_)_2_(Al_1-y_ , Sn_y_)C compositions synthesised in this study. The temperature refers to the synthesis temperature during reactive hot pressing.

Ceramic disks were synthesised via a powder metallurgical route. Starting powders of ZrH_2_ (<6 µm, Rockwood Lithium), NbH_0.89_ (< 40 µm, CBMM), Al (< 5 µm, AEE), Sn (< 5 µm, AEE) and C (< 5 µm, Asbury) were mixed in a near-stoichiometric ratio M:A:X = 2:1.1:0.95. A slight excess of the A-element was added to compensate for losses during sintering, while a substoichiometric amount of X compensated for the inward C diffusion from the graphitic sintering environment. In total 27 different ceramics were prepared with different Zr:Nb and Al:Sn ratios, as summarised in Fig. 1. Composition-wise, the produced ceramics can be divided in 4 groups: (1) M_2_AC ternary compounds that served as reference materials; (2) (Zr_1-x_, Nb_x_)_2_AlC and (3) Zr_2_(Al_1-y_ , Sn_y_)C quaternary compounds; and (4) the double solid solutions (Zr_1-x_, Nb_x_)_2_(Al_1-y_ , Sn_y_)C (quinary compounds). Since Zr-rich MAX phases are of primary interest, the amount of Nb in the double solid solution is limited to 20 at% on the M-site (i.e., x = 0.2).

The different powder mixtures were prepared in isopropanol using a multidirectional mixer (Turbula, WAB, Switzerland) for 24 h. The dried powders were pre-compacted at 30 MPa in a graphite die (Ø 30 mm) and densified by reactive hot-pressing (W100/150-2200-50 LAX, FCT Systeme, Frankenblick, Germany) in an actively maintained vacuum (10 Pa). The green powder compacts were heated at 20 °C/min under a load of 7 MPa up to the final synthesis temperature, which was composition-dependent, ranging from 1300 °C for Zr_2_SnC (~1250 °C)^17^, through 1500 °C for Zr_2_AlC (~1525 °C)^9^, to 1600 °C for Nb_2_AlC (1600 °C)^18^. Other temperatures were used for mixed compositions (i.e., with more than 3 elements). The sintering temperature for each composition is given in Fig. 1. Upon reaching the sintering temperature, the pressure was increased to 30 MPa and was maintained constant during a dwell time of 60 min. The M:A-ratio in the starting powder was adjusted to 2:1.05, in order to minimize the formation of intermetallic phases. After sintering, the surfaces of the ceramic discs were ground in order to remove the C-rich outer layer.

### Characterisation

X-ray diffraction (XRD) was used to identify and analyse the phase assembly in the sintered ceramic discs. The diffraction patterns were obtained using Cu *K*_α_ radiation in a Bragg-Brentano geometry. The diffractometer (Bruker D2) was operated at room temperature, at 30 kV and 10 mA. Measurements were performed in the 5° to 75° 2*θ* range with a step size of 0.02° 2*θ* and 0.2 s per step. Rietveld refinement was carried out using the Materials Analysis Using Diffraction (MAUD) software^19^. The *a* and *c* lattice parameters (LPs) were determined on the sintered discs with an accuracy of 10^−3^ Å and 5 × 10^−3^ Å, respectively. The line-broadening and size-strain model used for the refinement were based on the Popa rules^20^. The microstructure of mirror-polished ceramics was characterized with scanning electron microscopy (SEM; XL30-FEG, FEI, The Netherlands) equipped with an energy dispersive X-ray spectrometer (EDS; EDAX). Neutron powder diffraction (NPD) was carried out on Zr_2_SnC powder that was produced by ring milling (Retsch RS200) a piece of the respective hot-pressed disc during 1 min at 1000 rpm. The diffraction experiments were carried out with the KARL double axis diffractometer^21^, mounted on the Israeli Research Reactor 1. The measurement was performed using similar conditions as those used previously for Zr_2_AlC^9^. The NPD data were analyzed using the Rietveld refinement method, applied within the FullProf software.

## Results

### (Zr, Nb)_2_AlC

**Figure 2:**
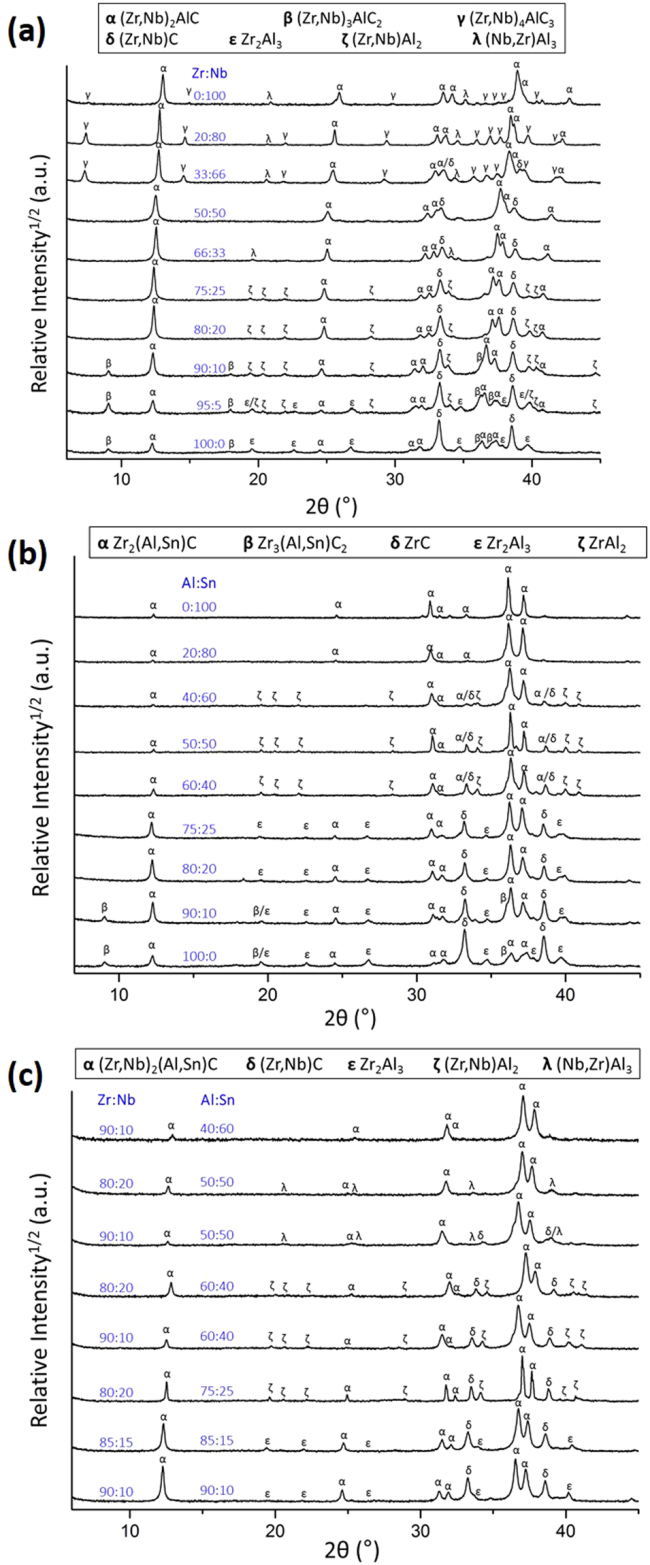
XRD patterns of the (**a**) (Zr_1-x_, Nb_x_)_2_Al_1.1_C_0.95_ ceramics, (**b**) Zr_2_(Al_1-y_ , Sn_y_)_1.1_C_0.95_ ceramics and (**c**) (Zr_1-x_, Nb_x_)_2_(Al_1-y_ , Sn_y_)_1.1_C_0.95_ double solid solution ceramics.

First, the effect of adding Nb as alloying element was investigated. Figure 2a shows the XRD patterns of ceramics with a (Zr_1-x_, Nb_x_)_2_Al_1.1_C_0.95_ starting composition (the refined XRD patterns are included in the Supporting Information). The main diffraction peaks of the constituent phases are identified, mainly corresponding to (Zr, Nb)_2_AlC and (Zr, Nb)C. The relative intensity of the (Zr, Nb)C peaks decreased with increasing Nb-content and this parasitic phase was completely absent at x = 0.8. At higher Nb-content (x ≥ 0.66), a fraction of (Nb, Zr)_4_AlC_3_ was observed, whereas the peaks of the 312 MAX phase could be identified at low Nb-content (x ≤ 0.1). Apart from the carbide phases, some secondary intermetallics were present, i.e., (Zr, Nb)Al_2_ and Zr_2_Al_3_ at low Nb-content and (Nb, Zr)Al_3_ in the Nb-rich ceramics. It is important to note that some of the parasitic phases can form (Zr, Nb)-based substitutional solid solutions. For example, (Zr_1-x_, Nb_x_)C has been reported as stable over the entire compositional range (0 ≤ x ≤ 1) above 570 °C^22^. The solubility ranges reported at 925 °C for (Zr_1-x_, Nb_x_)Al_2_ and (Nb_1-x_, Zr_x_)Al_3_ correspond to x = 0.15 and x = 0.68, respectively^23^. This indicates that alloying with Nb did not selectively favour the stabilization of the 211 MAX phase structure, and Nb was also incorporated in the competing parasitic phases.

**Figure 3:**
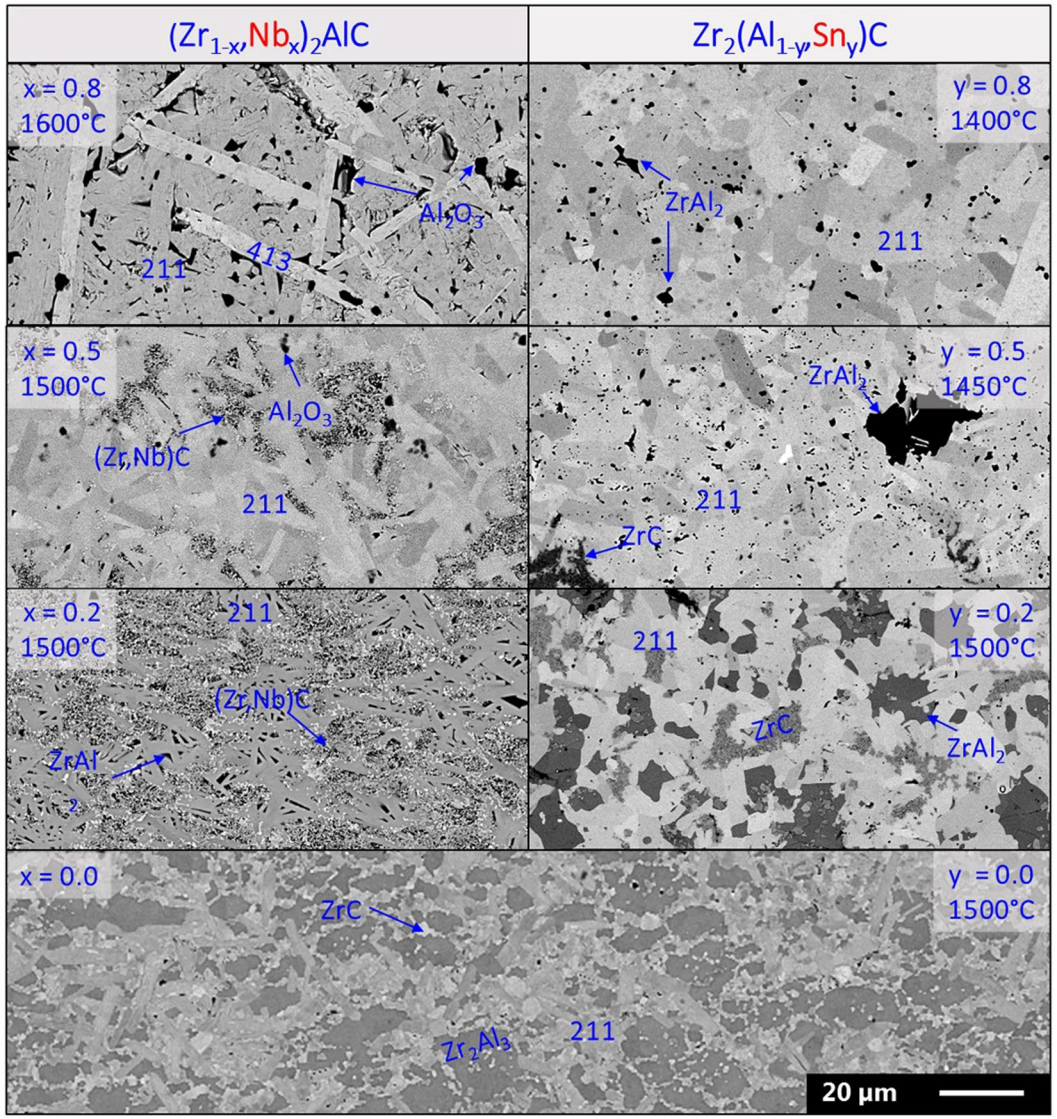
BSE micrographs of (Zr_1-x_, Nb_x_)_2_Al_1.1_C_0.95_ and Zr_2_(Al_1-y_ , Sn_y_)_1.1_C_0.95_ ceramics with varying Nb-content (left) and Sn-content (right). The scale bar in the lower right corner is valid for all micrographs.

The change in phase content was also reflected in the microstructure. The backscattered electron detector (BSE) micrographs of the (Zr_1-x_, Nb_x_)_2_AlC ceramics with different Nb-content (x = 0, 0.2, 0.5 and 0.8) are compared in the left column of Fig. 3. The elongated grains correspond to MAX phase grains, the volume fraction of which increased with increasing Nb-content. For x = 0, the relatively small grains of the 211 MAX phase are surrounded by Zr_2_Al_3_ regions with interspersed µm-sized ZrC particles. By adding Nb (x = 0.2 and 0.5), the (Zr, Nb)C grain size was significantly refined, a fact that could be attributed to a partial decomposition of the (Zr, Nb)_2_AlC into NbC and ZrC during cooling (<570 °C)^22^. Isolated Al_2_O_3_ particles were present at higher Nb-content (x = 0.5 and 0.8). These formed during the aluminothermal reduction of Nb_2_O_5_ that was present in the Nb-starting powder. Furthermore, 413 MAX phase grains can be distinguished at x = 0.8.

**Figure 4:**
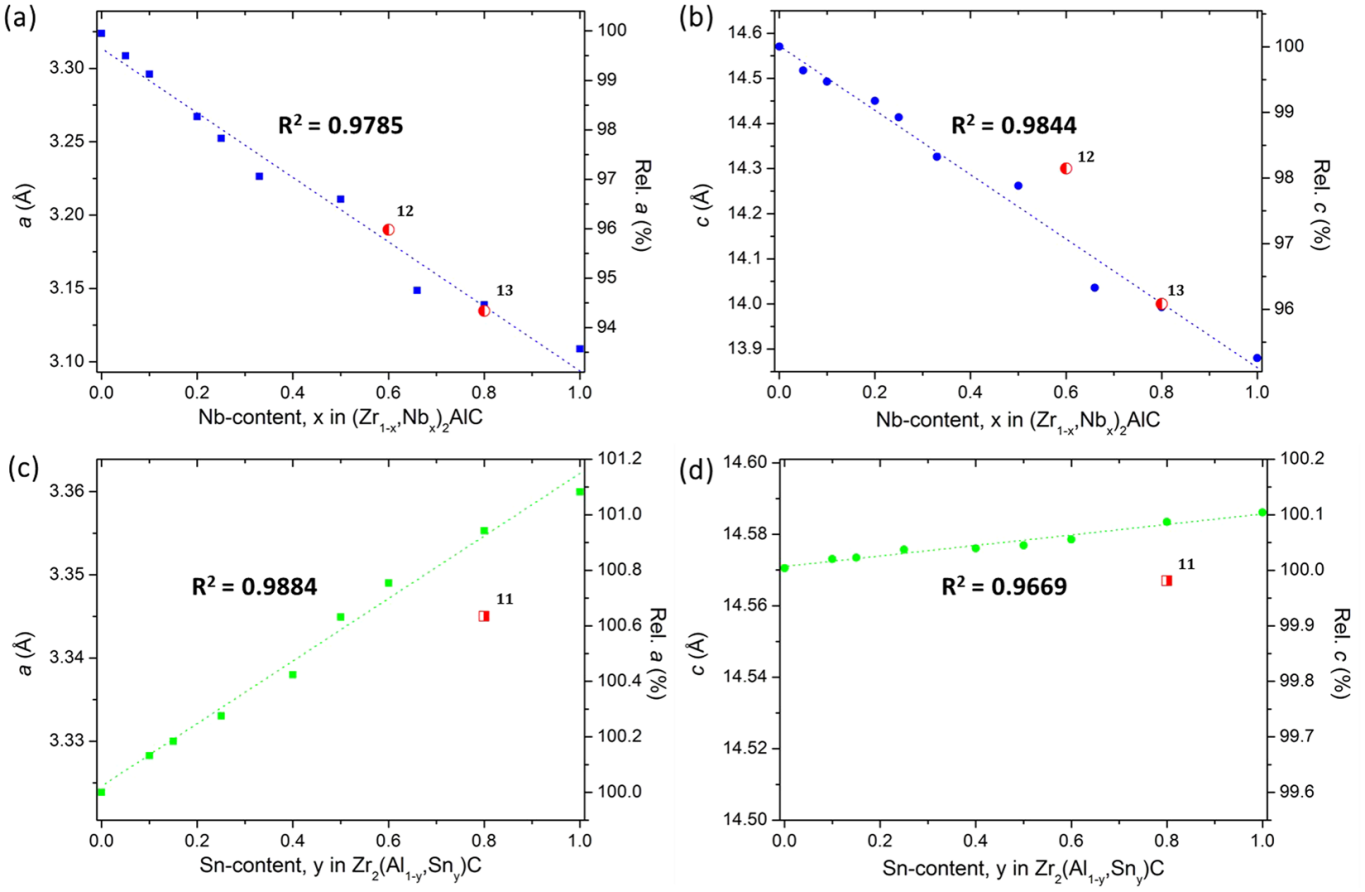
The evolution of the *a* and *c* LPs with the addition of Nb (**a**,**b**) and Sn (**c**,**d**).

The *a* and *c* LPs of (Zr, Nb)_2_AlC are shown in Fig. 4a,b, respectively, and some of them are included in Table 1 (blue squares). The relative value on the secondary (right) *y*-axis is a comparison with the LPs of Zr_2_AlC. A linear decrease of both *a* and *c* LPs with increasing Nb-content was observed resulting in a clear lattice shrinkage. This linear trend is in agreement with Vegard’s law, suggesting that (Zr, Nb)_2_AlC forms a solid solution over the entire compositional range. The shrinkage was caused by the smaller atomic radius of Nb as compared to Zr, an effect that was also observed in (Nb, Zr)_4_AlC_3_^24^. However, despite the fact that the starting powder was near-stoichiometric, synthesis of phase-pure materials was difficult as the rock-salt-structured (Zr, Nb)C was always present in the Zr-rich compositions, disappearing only at a high Nb-content (x = 0.8).

**Table 1:** A subset of the *a* and *c* LPs for the 211 phases in (Zr_1-x_, Nb_x_)_2_(Al_1-y_ , Sn_y_)_1.1_C ceramics, as determined from XRD (Fig. 2).

Sn-*y*	0			0.4			0.5			0.6		
Nb-*x*		*a (Å)*	*c (Å)*		*a (Å)*	*c (Å)*		*a (Å)*	*c (Å)*		*a (Å)*	*c (Å)*
0		3.324	14.571		3.338	14.576		3.345	14.577		3.349	14.579
0.1		3.296	14.508		3.316	14.518		3.324	14.523		3.327	14.527
0.2		3.267	14.451		3.290	14.460		3.295	14.464		—	—

The uncertainty on the experimentally obtained LPs is in the range of ±10^−3^ Å. The coloured symbols correspond to those used in Fig. 1.

Two reports on (Zr, Nb)_2_AlC are available in literature. In 1966, Reiffenstein could substitute Nb by Zr in a Nb/Zr ratio of 1.5 or equivalently synthesise (Zr_0.4_, Nb_0,6_)_2_AlC, starting from Nb_2_AlC^12^. However, no further information on the starting composition or additional phases was provided. More recently, Naguib reported on various solid solutions and the formation of (Zr_0.2_, Nb_0.8_)_2_AlC, starting from a Zr:Nb ratio of 25:75, with about 9 wt% of ZrC and a minor amount of Zr_5_Al_3_^13^. The reported LPs are included in red in Fig. 4a,b and fit the suggested trend line. The outlier for the *c*-value of (Zr_0.4_, Nb_0.6_)_2_AlC might be attributed to its large uncertainty, which was reported to be 14.3 ± 0.7 *Å*^12^. Overall, it is clear that the addition of Nb promotes the formation of the 211 MAX phase in the Zr-Nb-Al-C system and lowers the relative stability of (Zr, Nb)C as compared with the (Zr, Nb)_2_AlC phase. However, the required Nb-content to obtain phase-pure MAX phase materials is high (x > 0.66).

### Zr_2_(Al, Sn)C

Second, the effect of adding Sn as alloying element was investigated and Fig. 2b shows the XRD patterns of the produced Zr_2_(Al_1-y_ , Sn_y_)_1.1_C_0.95_ ceramics (the refined XRD patterns are included in the Supporting Information). Similarly as in the addition of Nb, the rock-salt-structured ZrC was the main competing phase. A small fraction of the 312 MAX phase was observed at low Sn-content (y ≤ 0.1). For y ≤ 0.25, the Zr_2_Al_3_ intermetallic compound was detected, whereas ZrAl_2_ was found in the ceramics with higher Sn-content. In contrast with the Nb substitution, the competing phases do not form a notable solid solution with Sn and, thus, this alloying element was selectively incorporated into Zr_2_(Al, Sn)C. This results in an effective stabilization of the 211 MAX phase, which was nearly phase-pure at y = 0.6, with 4 wt% ZrC and 9 wt% ZrAl_2_. ZrAl_2_ was mainly present due to the superstoichiometric amounts of A-elements (Al and Sn) in the starting powder (M:A = 2:1.1), which resulted in the formation of a Zr_2_(Al_0.34_, Sn_0.66_)C solid solution, as Sn was exclusively dissolved in the MAX phase and the leftover Al formed ZrAl_2_. The XRD patterns were used to calculate the LPs, the evolution of *a* and *c* with the Sn-content in the starting powder is shown in Fig. 4c,d, respectively, and some of the data are included in Table 1 (green dots). A good fit with the linear trend of Vegard’s law was obtained by assuming that Sn can substitute Al over the entire compositional range. The *a* values for the high-Sn-containing ceramics (y = 0.5, 0.6 and 0.8) were slightly above the linear trend, due to the superstoichiometric ratio of A in the starting powder, and the resulting MAX phase composition corresponds roughly to Zr_2_(Al_0.45_, Sn_0.55_)C, Zr_2_(Al_0.34_, Sn_0.66_)C and Zr_2_(Al_0.12_, Sn_0.88_)C, respectively. The substitution of Sn caused an expansion of the unit cell, mainly due to an increase in the *a*-LP, whereas *c* remained almost constant with only a relative difference of 0.1% between Zr_2_AlC and Zr_2_SnC. This was also observed by Horlait *et al*. for Zr_2_(Al, A)C with A = Sn and other heavy A-elements, such as Pb, Sb and Bi^11^. The values reported for Zr_2_(Al_0.2_, Sn_0.8_)C are included in Fig. 4c,d. Both parameters are lower compared to the observed trend in this study. However, the differences are small, i.e., <0.4% for *a* and <0.05% for *c*, which can be attributed to local variations in composition.

The microstructure of the produced Zr_2_(Al_1-y_ , Sn_y_)_1.1_C_0.95_ ceramics for y = 0, 0.2, 0.5 and 0.8 is shown in the right column of Fig. 3. The three constituent phase types are indicated on the images. With increasing Sn-content, the ZrC and intermetallic phase (Zr_2_Al_3_ and ZrAl_2_) fractions clearly decreased. These intermetallics are present as separate patches between the MAX phase grains. This morphology differs from the (Zr_1-x_, Nb_x_)_2_AlC microstructure, where the intermetallics mainly act as binder for the finely dispersed (Zr, Nb)C. Also the morphology of the MAX grains changed with the added element. The platelet shape was more pronounced when Nb was added, whereas the addition of Sn resulted in more equiaxed grains. It is inferred that the observed difference in anisotropic grain growth reflects differences in the *c*/*a*-ratio, which decreased from 4.465 for Nb_2_AlC, through 4.384 for Zr_2_AlC, to 4.341 for Zr_2_SnC.

### (Zr, Nb)_2_(Al, Sn)C

Thirdly, the effect of adding both Nb and Sn was investigated and the XRD patterns of the 8 produced (Zr_1-x_, Nb_x_)_2_(Al_1-y_ , Sn_y_)_1.1_C_0.95_ ceramics are compared in Fig. 2c (the refined XRD patterns are included in the Supporting Information). The amount of Nb added to the double solid solutions was limited to x ≤ 0.2 for three reasons: (1) the main interest is in Zr-based MAX phases due to their small neutron cross-section, (2) taking into account the observations made for the two pseudo-ternary systems, where Nb can be incorporated in the competing phases while Sn is selectively only present in the 211 MAX phase, and (3) Nb-rich MAX phases are known to have a poor oxidation resistance^25^.

**Figure 5:**
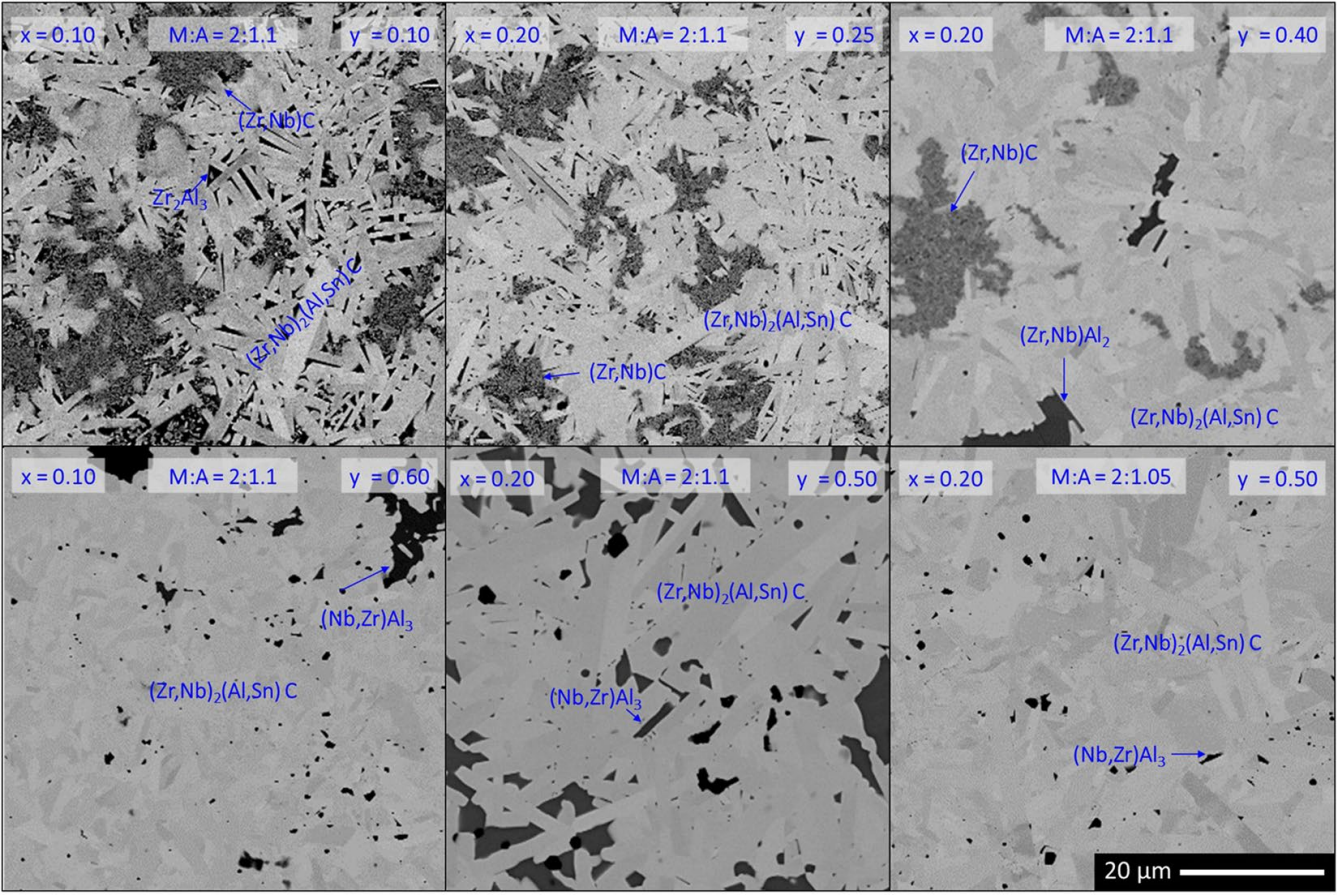
BSE micrographs of various (Zr_1-x_, Nb_x_)_2_(Al_1-y_ , Sn_y_)C-based double solid solutions. The scale bar in the lower right corner is valid for all micrographs.

Analysing the evolution of the phase content, a similar trend as for the quaternary systems was found. Adding Nb and Sn to the Zr-Al-C system increased the fraction of the 211 MAX phase and no parasitic (Zr, Nb)C phase was observed for the combinations of Zr:Nb & Al:Sn equal to 80:20 & 50:50 and 90:10 & 40:60. This observation is supported by the microstructural evolution, as shown in Fig. 5. At lower Sn substitution levels, (Zr, Nb)C was always present with an intermetallic compound to accommodate the Al surplus. The intermetallic compound evolved with increasing Sn-content from Zr_2_Al_3_ (y ≤ 0.15), through (Zr, Nb)Al_2_ (0.25 ≤ y ≤ 0.4), to (Nb, Zr)Al_3_ (y = 0.5).

In order to avoid the formation of these intermetallic phases, the M:A-ratio in the starting powder was adjusted from 2:1.10 to 2:1.05 at a later stage of this study. This difference in M:A-ratio is indeed clearly reflected in the microstructure of the two ceramics with x = 0.2 and y = 0.5 shown in Fig. 5. The *a* and *c* LPs for some of the double solid solutions are included in Table 1 (red triangles). The trends for the *a* and *c* LPs are the same as those observed in the quaternary systems. An increase in Sn-content results mainly in an increased *a*-LP, whereas *c* increases only slightly. The addition of Nb decreases both LPs. These trends are further investigated in the next section, which attempts to correlate the *a* and *c* LPs with the stability of the 211 MAX phases.

## Discussion

**Figure 6:**
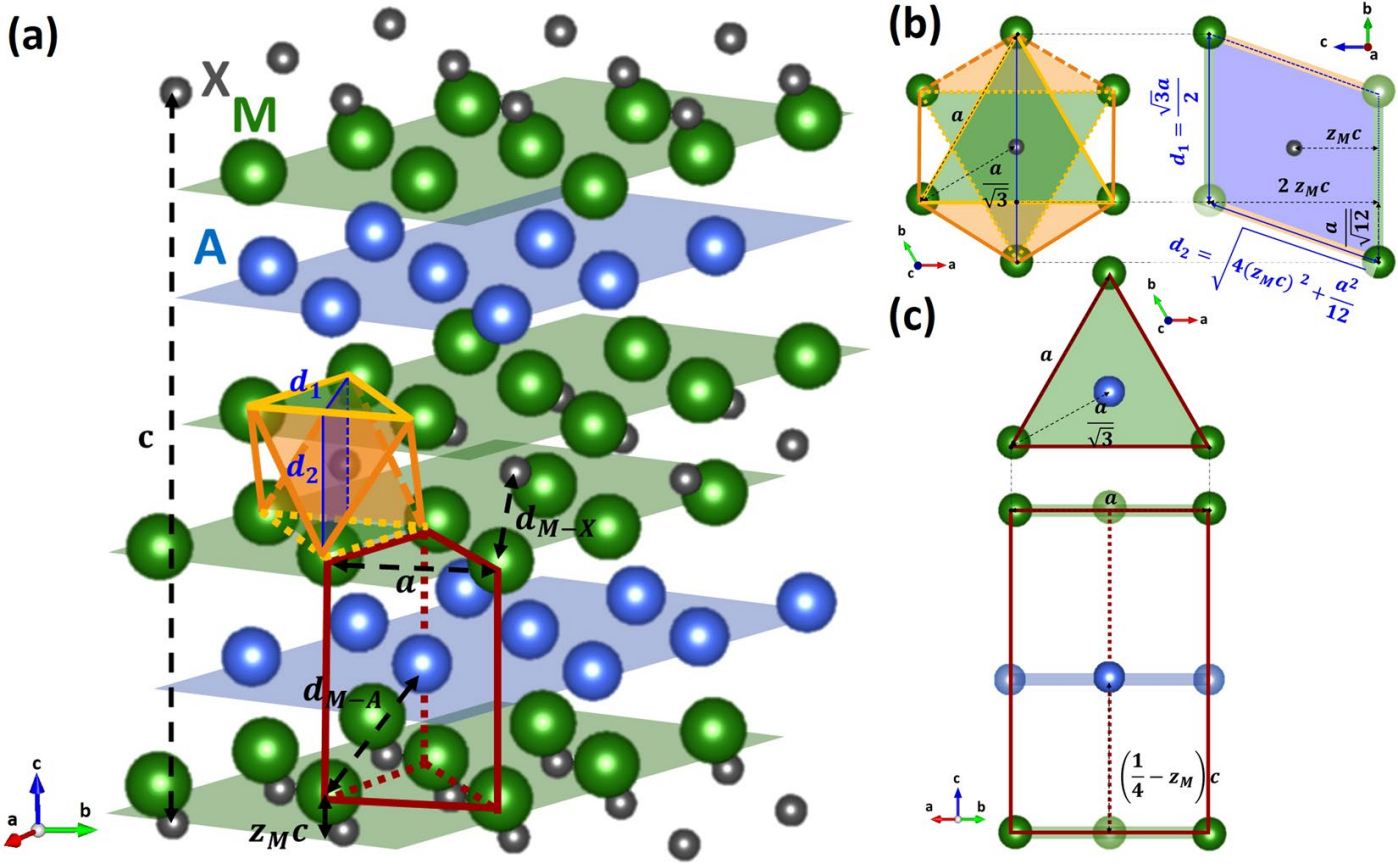
The crystallographic parameters as defined for M_2_AX with (**a**) the 3D-structure with the M_6_X octahedron indicated in orange and the M_6_A trigonal prism indicated in red; (**b**) the projected octahedron; and (**c**) the projected trigonal prism.

It is clear that the addition of Nb and/or Sn to the starting powder increases the phase stability of the 211 MAX phases, as compared to the stability of the competing binary carbide ZrC. The reason for this, as inspired by Hägg’s rule for binary carbides^26^, might be found in the way these elements alter the crystallographic parameters and concomitantly sterically stabilize the MAX phase structure. In the M_2_AX phase structure (Fig. 6a), there are 3 free variables determining the unit cell: *a*, *c* and the relative z-coordinate of the M-atom, z_M_. The latter defines the height of the M-atoms above the layer of C or N, and varies commonly in the 0.08–0.09 range. Obviously, z_M_ directly affects the distance between the M and X atoms, *d*_*M−X*_:

1 $${d}_{M-X}=\sqrt{\frac{{a}^{2}}{3}+{(c{z}_{M})}^{2}}$$

As may be seen from the schematic representation of the 211 MAX phase structure in Fig. 6a, the unit cell can be considered as an alternating stack of M_6_X-octahedra, indicated in orange, and M_6_A-triangular prisms, indicated in red. A similar representation of the crystal structure is valid for the other common MAX phase types, 312 and 413, with a building block of 2 and 3 M_6_X-octahedra interleaving the M_6_A-layers, respectively.

For the binary NaCl-structured MX phase, the unit block consists of a cubic octahedron with fourfold symmetry axes. In the case of M_2_AX, however, the octahedral block is not ideally cubic, resulting in a reduced symmetry. This distortion can be quantified by the distortion parameter, *o*_*d*_, which is the ratio of distances between two octahedral faces off the basal plane (d_1_) and two octahedral faces in the basal plane (d_2_)^27^. These distances are indicated in Fig. 6a and are measured on the octahedral faces, as defined by the projections in Fig. 6b. The ratio corresponds to^27^:

2 $${o}_{d}=\frac{{d}_{1}}{{d}_{2}}=\frac{\sqrt{3}}{2\sqrt{4{{z}_{M}}^{2}{(\frac{c}{a})}^{2}+\frac{1}{12}}}$$

In the 211 MAX phase structure, the distortion is influenced by *c*/*a* and z_M_. *o*_*d*_ becomes equal to 1 for a perfect cubic octahedron, which would assume ideal, close-packed spheres $(c/a=2\sqrt{6\,}\approx 4.89)$ and the M-atom at the canonical position (*z*_*M*_ = 1/12 ≈ 0.0833). Typically, the values reported for *o*_*d*_ are >1, indicating a compression of the octahedron block along the *c*-axis.

Similarly, a parameter is defined for the distortion of the trigonal prism, *p*_*d*_, which is expressed as the ratio of the distance between the M-atoms in the basal plane, *d*_*M−M*_(*d*_*M*−*M*_ = *a*), and the distance between M- and A-atoms, *d*_*M−A*_^28,29^. The latter can be calculated from the projections in Fig. 6c, and corresponds to:

3 $${d}_{M-A}=\sqrt{\frac{{a}^{2}}{3}+{c}^{2}{(\frac{1}{4}-{z}_{M})}^{2}}$$

The distortion of the trigonal prism can be calculated as follows^28,29^:

4 $${p}_{d}=\frac{{d}_{M-M}}{{d}_{M-A}}=\frac{1}{\sqrt{\frac{1}{3}+{(\frac{1}{4}-{z}_{M})}^{2}{(c/a)}^{2}}}$$

Similar to *o*_*d*_, *p*_*d*_ equals 1 for an ideal packing of hard spheres and depends only on the crystallographic parameters *c*/*a* and z_M_. Commonly, *p*_*d*_ > 1 pd, which corresponds to a compression along the *c*-axis as well.

These distortion parameters have been used in density functional theory (DFT) calculations to determine the free crystallographic parameters. Kanoun *et al*. calculated the effect of the A-element in Zr_2_AC, reporting that distortions *o*_*d*_ and *p*_*d*_ increased with the number of valence electrons and with the trigonal prism becoming more strongly affected^30^. Moreover, they studied the effect of the M-element in M_2_SnC with M = Ti, Zr, Hf and Nb, reporting a lower distortion for the octahedral block of Zr_2_SnC and Hf_2_SnC, as compared to the Ti- and Nb-containing M_2_SnC. They reported this difference as a ‘steric effect’ and correlated it with the size of the M-atom^31^. Also Horlait *et al*. suggested a kind of steric effect in terms of the *a-* and *c*-ranges in which these 211 structures might be stable^11^. This suggestion is based on the earlier observation that the A-element mainly alters the *a*-LP, hence, modifying the *c*/*a* ratio.

Coming back to the distortion parameters, the A-element also modifies *o*_*d*_ and *p*_*d*_. Therefore, it is interesting to see how these distortion parameters vary with varying solute content in the studied solid solutions. One of the main challenges to quantify these parameters, however, is the limited availability of accurate, experimentally measured literature data on z_M_. Some experimentally obtained *c*/*a* and z_M_ values for ternary MAX phases are listed in Table 2, together with the calculated crystallographic parameters according to Eqs (1–4).

**Table 2:** Experimentally determined crystallographic parameters of some ternary M_2_AC phases.

MAX Phase	*c*/*a*	z_M_	*d*_*M−A*_	*d*_*M−X*_	*o*_*d*_	*p*_*d*_	Method	Ref.
*ideal hcp spheres*	$2\sqrt{6\,}\,\approx 4.89$	1/12 ${\rm{\approx }}0.0833$	*—*	*—*	*1*	*1*	*—*	*—*
Ti_2_GaC	4.349	0.0865	2.817	2.120	1.074	1.092	SC-XRD	^33^
Cr_2_GaC	4.355	0.0853	2.671	1.991	1.087	1.086	SC-XRD	
Cr_2_GeC	4.101	0.0845	2.631	1.988	1.153	1.122	XRD	^32^
Cr_2_AlC	4.478	0.0865	2.669	1.990	1.048	1.072	XRD	
	4.480	0.0855	2.681	1.984	1.058	1.068	XRD	^44^
V_2_AlC	4.501	0.0856	2.732	2.020	1.053	1.0653	SC-XRD	^34^
Ti_2_AlC	4.461	0.0850	2.861	2.112	1.067	1.072	EXAFS	^27^
Nb_2_AlC	4.471	0.0883	2.874	2.172	1.031	1.081	EXAFS	
Hf_2_AlC	4.204	0.0877	2.928	2.244	1.094	1.108	XRD	^37^
Zr_2_AlC	4.384	0.0871	3.052	2.301	1.061	1.089	XRD	^9^
	4.379	0.0898	3.020	2.322	1.034	1.101	NPD	
Zr_2_SnC	4.359	0.0860	3.076	2.304	1.078	1.088	XRD	^2^
	4.341	0.0857	3.083	2.308	1.085	1.090	XRD	This work
	4.337	0.0861	3.076	2.310	1.082	1.092	NPD	

The atomic distances and distortions are calculated according to Eqs (1–4).

In order to estimate *o*_*d*_ and *p*_*d*_ for solid solutions, one must differentiate between the effect of M- and A-site solid solutions. Hug *et al*. determined the z_M_ of Ti and Nb in the M-site solid solution (Ti_0.5_, Nb_0.5_)_2_AlC, using extended X-ray absorption fine structures (EXAFS). They noticed a different atomic position for Ti and Nb with z_Ti_ and z_Nb_ values close to those observed for Ti_2_AlC and Nb_2_AlC, respectively^27^. This suggests that the addition/substitution of an M-element only modifies slightly the atomic position of the host M-element.

With respect to A-site solid solutions, Cabioc’h *et al*. determined z_Cr_ in Cr_2_(Al, Ge)C using XRD. Notwithstanding the large statistical error, they reported a linear trend for the evolution of z_Cr_ between Cr_2_AlC and Cr_2_GeC. Concomitant with a linear evolution of the *a* and *c* LPs, following Vegard’s law, a gradual decrease of *o*_*d*_ and *p*_*d*_ was observed with increasing Al-content^32^. A similar correlation can be established for z_V_ combining the reports of Etzkorn *et al*., who determined the LPs of V_2_(Al_1-y_ , Ga_y_)C with 1 ≥ y ≥ 0.43, using single crystal X-ray diffraction (SC-XRD)^33,34^. These reports indicate that the distortion parameters can be tuned by modifying the composition of the MAX phase, especially on the A-site.

Following this reasoning, the crystallographic parameters of Zr_2_SnC are of particular interest, as they can be used in combination with the z_M_ value for Zr_2_AlC to estimate the distortion parameters for Zr_2_(Al, Sn)C. Jeitschko *et al*. reported on the experimentally obtained *a* and *c* values and gave a generic value for z_M_, valid for most reported H-phases^2^. Barsoum *et al*. reported on the experimentally obtained *a* and *c* values for M-Sn-C phases, but no information on z_zr_ was included^17^. In order to have a reliable and direct comparison with the z_zr_ value for Zr_2_AlC^9^, neutron diffraction analysis was performed on Zr_2_SnC powder (the refined pattern is included in the Supporting Information). This characterization technique is more precise in determining the crystallographic structure; therefore, the crystallographic parameters, especially z_M_, are considered to be more accurate. The neutron diffraction data of *c*/*a* and z_Zr_ are included in Table 2. They are in good agreement with the XRD values obtained in this study and differ only slightly from the data reported by Jeitschko *et al*.^2^. Moreover, all three data sets for Zr_2_SnC show a similar trend in comparison with Zr_2_AlC, i.e., both *c*/*a* and z_Zr_ decreased. The calculated distortion parameters for the octahedral blocks and trigonal prisms are included in Table 2.

Of particular interest are the values for Zr_2_AlC. The distortion of the trigonal prism for this compound is significantly larger than for most other M_2_AlC (except for Hf_2_AlC). Replacing Al by Sn lowers the *p*_*d*_, as can be concluded from the NPD results of Zr_2_SnC. The decreased z_M_ was more determining than the decreased *c*/*a*–ratio. Moreover, replacing Zr with Nb also caused a decrease in *p*_*d*_, as z_M_ is lower in Nb_2_AlC and the *c*/*a*-ratio is larger (based on the EXAFS results in^27^).

**Figure 7:**
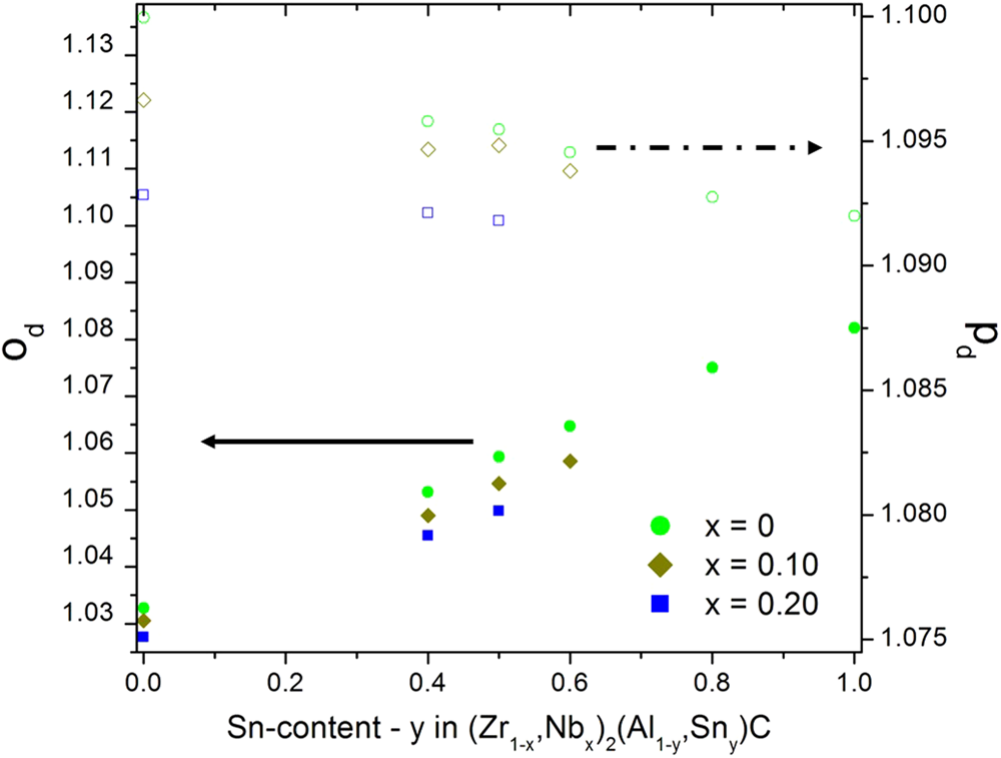
The distortion parameters *o*_*d*_ (solid symbols) and *p*_*d*_ (open symbols) as a function of the Sn-content for solid solutions with variable Nb-content.

Based on the preceding discussion, it finally becomes possible to combine these values for the ternary compounds with the above mentioned trends observed in M- and A-site solid solution formation. The z_M_ values for the solid solutions are assumed to be a linear interpolation of the ternary end-members listed in Table 2. The trends for the distortion parameters of the (Zr, Nb)_2_(Al, Sn)C double solid solution are illustrated in Fig. 7. Overall, the double solid solutions are characterised by a smaller distortion of the trigonal prism as compared to Zr_2_AlC. Both substituting elements, Nb and Sn, contribute to a reduction in *p*_*d*_. On the other hand, *o*_*d*_ increases significantly with increasing Sn-content, whereas Nb does not alter significantly the octahedral distortion. This analysis of the distortion parameters can be extended to the other values obtained for M_2_AC phases in Table 2. Before further evaluating these numbers, it should be emphasised that the *c*/*a* and z_M_ data were determined with different characterization techniques and were collected from different published works. Therefore, a direct comparison is neither ideal nor recommended. Nevertheless, in the absence of a set of consistently measured z_M_ data for M_2_AC phases, the available values can provide a first insight into the issue at hand.

With respect to the values in Table 2, Cr_2_GeC has clearly the largest distortions for both building blocks. This is mainly due to the small *c*/*a* ratio of this MAX phase compound, as the distortions increase with decreasing *c*/*a*, according to Eqs (2–4). The low z_M_ value is the reason why *o*_*d*_ > *p*_*d*_, as the former is inversely proportional to z_M_, whereas the latter scales with the z-coordinate of the M-atom. Considering these large distortion parameters for Cr_2_GeC, a stable M_2_AC compound, we are not able to specify a range of numbers associated with crystal geometry that unambiguously determines phase stability.

**Figure 8:**
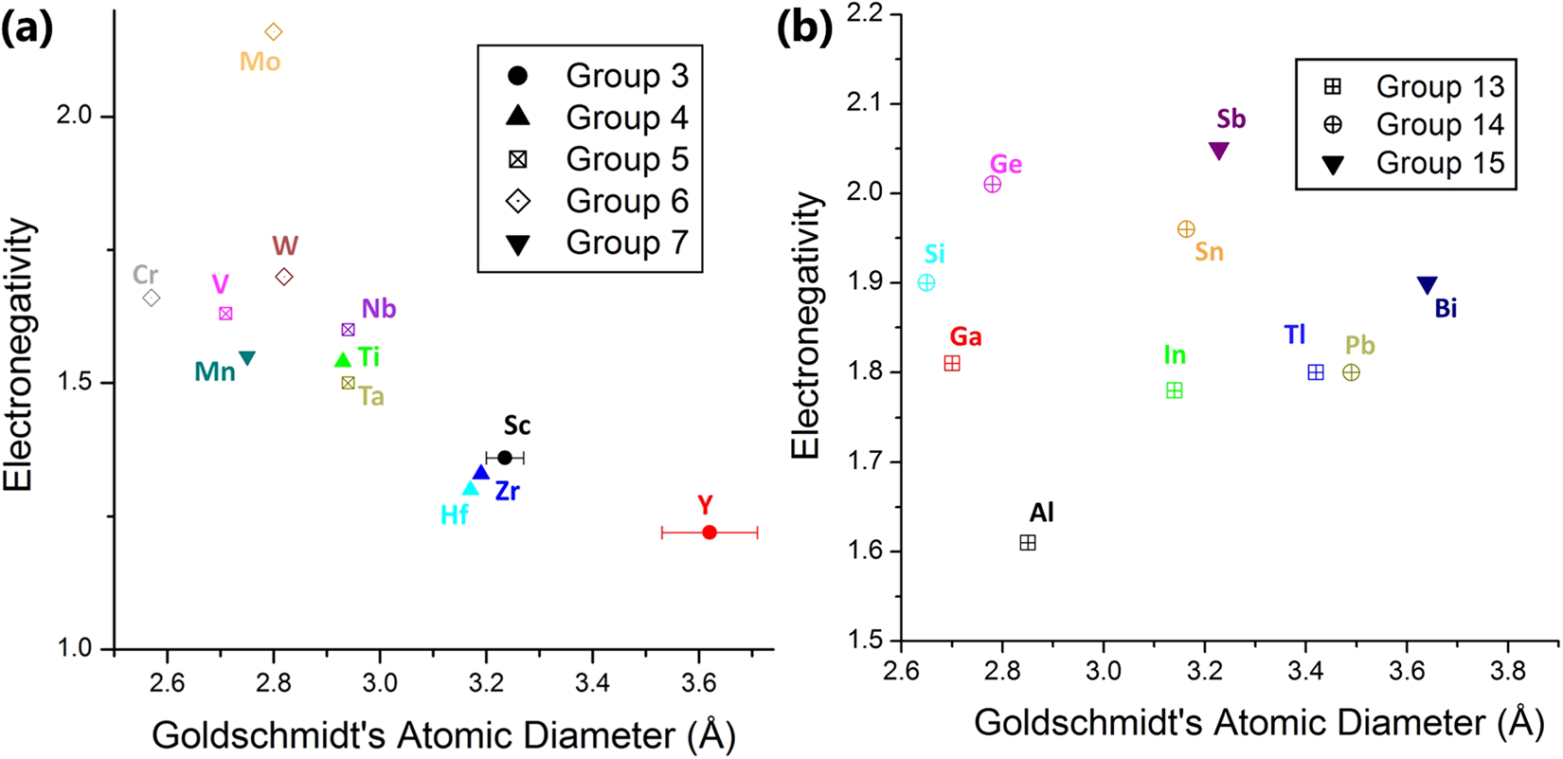
Goldschmidt’s atomic diameter vs. electronegativity for different (**a**) M-metals and (**b**) metallic A-elements. The atomic diameter data were obtained from^35^.

However, the distortion of the trigonal prism is an interesting case, as it serves as a measure for the steric match between M- and A-elements, where *p*_*d*_ = 1 means that M and A are identical. From that perspective, and in accordance with Hägg’s rule for binary carbides, it is worthwhile comparing the atomic radii of different M- and A-elements. An overview of Goldschmidt’s atomic diameter^35^ plotted as function of electronegativity is given in Fig. 8. For M_2_AlC compounds, the largest *p*_*d*_-values are found for Zr and Hf, which have similar atomic radii, and are the two largest M-atoms, outsized only by the rare-earth elements Sc and Y (Fig. 8a). On the other hand, Al is one of the smaller A-atoms based on Fig. 8b. Therefore, a large *p*_*d*_ value can be interpreted as a large steric mismatch associated with differences in the size (atomic radii) of the M- and A-elements.

**Figure 9:**
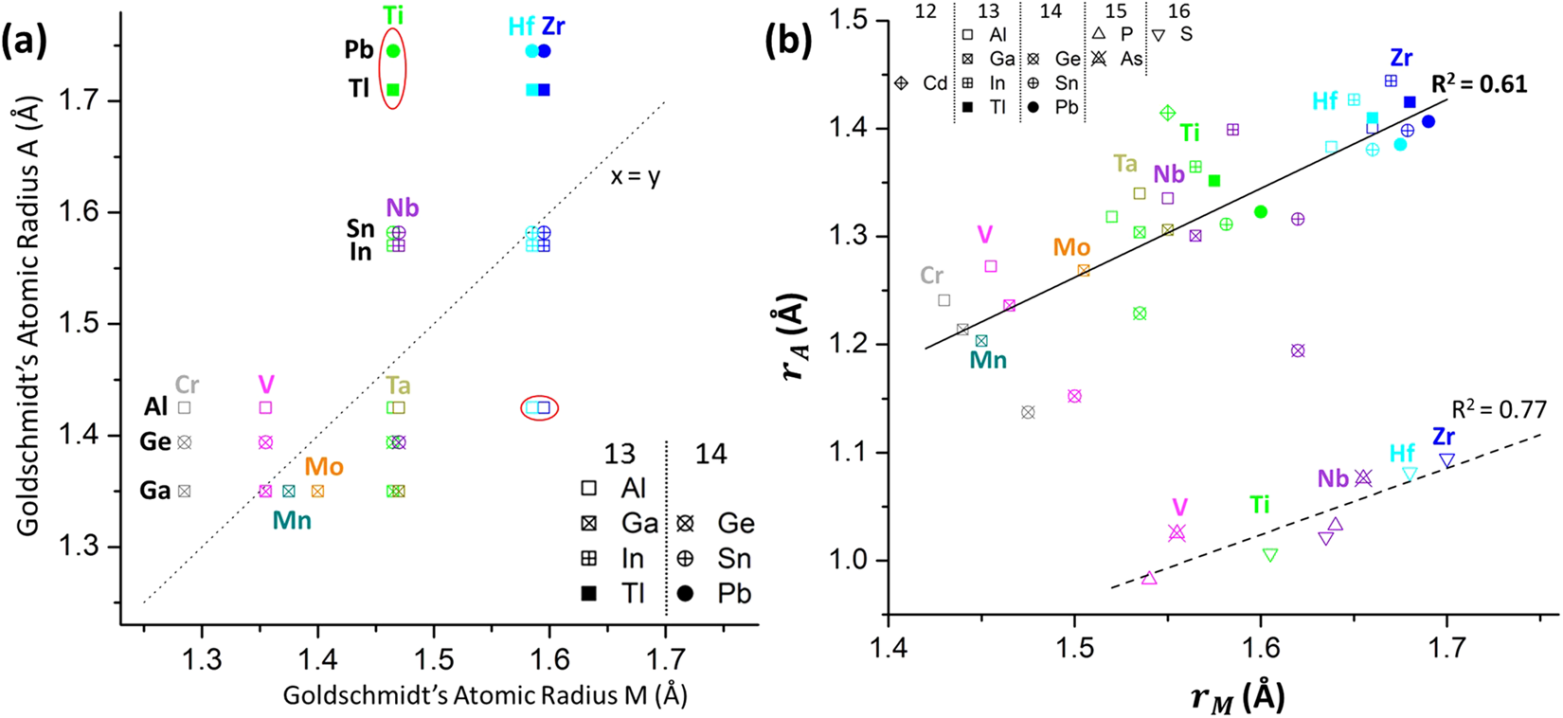
Comparison of the atomic radii of the M- and A-elements in M_2_AC MAX phases, based on (**a**) Goldschmidt’s atomic radii, and (**b**) the radii values determined by Eqs (5) and (6). The red ellipses indicate the outliers discussed in the text.

A steric point of view on the reported, experimentally synthesized M_2_AC structures is obtained when the atomic radii of the M-elements are plotted versus the radii of the A-elements (from groups 13 and 14). This elementary plot is shown in Fig. 9a. It appears that, overall, large M-atoms preferentially combine with large A-atoms, and the same holds for the small M- and A-elements. This possibly explains why certain M/A combinations are not observed experimentally. However, some outlier combinations have been reported, i.e., Zr_2_AlC, Hf_2_AlC, Ti_2_PbC and Ti_2_TlC (indicated by red ellipses in Fig. 9a). These phases can be regarded on the cusp of steric stability. MAX phases in the Zr-Al-C and Hf-Al-C systems combine a large M-atom with a small A-atom. They have been only recently experimentally synthesised^9,36,37^, always in combination with MC binary carbides and never as phase-pure bulk compounds. This might be associated with the aforementioned steric effect and their outlier positions in Fig. 9a. A similar comment can be given for both Ti_2_PbC and Ti_2_TlC, which combine a small M-atom with a large A-atom. These compounds were discovered by Jeitschko *et al*. in 1964 and diverted significantly from the conventional H-phase structure. A first oddity is the value of z_Ti_ in these compounds, as it was specifically discussed and lowered to 0.08 instead of 0.086 for the other H-phases. Moreover, the Pb-defect structure for Ti_2_PbC was described as ‘(*Ti*_*1.97*_, *Pb*_*0.03*_)_2_(*Pb*_*0.9*_, *Ti*_*0.03*_)*C*_*1-x*_’ and a similar structure was assumed for the Tl-containing equivalent (*‘Ähnliches gilt auch für die Phase Ti*_*2*_*TlC’*)^38^. Curiously, no other reports on these H-phases (members of the MAX phase family) were found thereafter.

Figure 9b is an alternative plot for all 40 M_2_AC phases, which shows the atomic radii of M (*r*_*M*_) versus the atomic radii of A (*r*_*A*_), based on their size in the MAX phase lattice, with:

5 $${r}_{M}=\frac{a}{2}$$

and

6 $${r}_{A}={d}_{M-A}-{r}_{M}=\sqrt{\frac{{a}^{2}}{3}+{c}^{2}{(\frac{1}{4}-{z}_{M})}^{2}}-\frac{a}{2}$$

where z_M_ is generically chosen equal to 0.086. In Fig. 9b, two groups can be clearly distinguished. The points in the lower part of the plot correspond to the 7 MAX phases based on P, S and As, whereas the points in the upper plot part correspond to the other 33 M_2_AC MAX phases. For both groups, a linear correlation between *r*_*M*_ and *r*_*A*_ can be postulated, where the values for *r*_*M*_ are in general larger than *r*_*A*_. The significantly smaller *r*_*A*_ for the MAX phases containing elements from groups 15 and 16 (non-metallic and metalloid elements) is attributed to their small *c*/*a* ratio. The latter might be related with the large electronegativity value of P (2.19), S (2.58) and As (2.18), as compared to the metallic A-elements (cf. Fig. 8b). The deviatory behaviour of S, and to a lesser extend of As, was also noticed by Barsoum when plotting the number of valence electrons (n_val_) versus the theoretical density of states at the Fermi level (N(E_f_))^29^. No P-containing phases were included there. One additional remark, the atomic radii for the MAX phases containing the metalloid Ge (2.01) are found between the two linear trends, indicating that the non-metal/metal transition of the periodic table can also be observed in the MAX phase structure.

Coming back to the initial impetus of this work, i.e., the synthesis of a phase-pure Zr_2_AlC-based MAX phase solid solution, one can interpret the effect of the different solute atoms in a steric way. The addition of Nb reduces the size of the average M-atom, whereas the addition of Sn increases the average size of the A-atom. In this way, the additives reduce the steric mismatch in the trigonal prism. This suggestion closely relates with one of the guidelines for solid solution formation in metallic alloys, formulated by Hume-Rothery^35^. This steric interpretation also provides an explanation for the trend that Horlait et al. observed for Zr_2_(Al, A)C solid solutions, i.e., that solute elements larger than Al, i.e., Sn, Pb, Sb and Bi, facilitated the formation of the Zr-containing 211 MAX phase^11,16^.

The Hume-Rothery atomic radii rule also holds for most reported (M, M′)_n+1_AC_n_ and M_n+1_(A, A′)C_n_ solid solutions, where a more limited solubility is observed with increasing difference in atomic radii^13,39,40^. As case study, the literature on Cr_2_AC-based MAX phases is investigated, with Cr the smallest M-element, in contrast to Zr that is the largest M-element according to Fig. 8b. The three ternary systems experimentally reported are Cr_2_AlC, Cr_2_GeC and Cr_2_GaC. These A-elements are small atoms that can replace each other resulting in the reported solid solutions Cr_2_(Al, Ge)C^32^ and Cr_2_(Al, Ga)C^33^, in agreement with the Hume-Rothery rule. A third solid solution is reported, i.e., Cr_2_(Al, Si)C^41^. Even though no ternary M_2_SiX phase exists to the best of our knowledge, the only 211 structured solid solution containing Si, the smallest reported A-element according to Fig. 8b, forms in combination with Cr, the smallest reported M-atom.

An additional remark with respect to the atomic radius match is that when M/A and M′/A′ differ significantly, substitution might be very limited or, alternatively, in-plane ordering of the atoms might occur. The latter is illustrated by the recently synthesised, in-plane ordered 211 “i-MAX” phases, i.e., (Mo_2/3_, Sc_1/3_)_2_AlC, (V_2/3_, Zr_1/3_)_2_AlC, (Cr_2/3_, Sc_1/3_)_2_AlC, (Cr_2/3_, Y_1/3_)_2_AlC and (Mo_2/3_, Y_1/3_)_2_AlC^15,42,43^, where M:M′ = 2:1 and the atomic radius of M is substantially smaller than that of M′ (see Fig. 8a).

To conclude this section, there seems to be a steric effect present in the M_2_AC structure that can be used to improve the stability and phase purity of certain M-A-C combinations. The proposed rule of thumb implicates the partial substitution of the M- and/or A-element with an element that is appropriate in terms of atomic radius. A good match between the atomic radii of M and A should be targeted in agreement with the graphs of Fig. 8. In order to further validate this suggestion, however, more data on experimentally obtained solid solutions are required. In a second step, it would be possible to further elaborate the correlation of phase stability with the lattice distortion parameters. In order to do so, accurately determined z_M_ parameters for the various ternary and quaternary MAX phase systems are required.

## Conclusions

The possibility to stabilize Zr_2_AlC and to eliminate parasitic ZrC formation by the addition of Nb and/or Sn was investigated. The two quaternary systems (Zr, Nb)_2_AlC and Zr_2_(Al, Sn)C were found to be stable over their entire compositional range and both Nb and Sn promoted the formation of the 211 MAX phase. Sn is preferred over Nb, as it is selectively incorporated into the MAX phase structure, whereas Nb is also observed in most competing phases, i.e., (Zr, Nb)C, (Zr, Nb)Al_2_ and (Nb, Zr)Al_3_. The lattice parameters (LPs) *a* and *c* were determined by XRD for both solid solutions and obeyed Vegard’s law. The addition of Nb significantly lowered both LPs, whereas the substitution of Al with Sn mainly increased the *a*-LP and left *c* practically unaffected. A similar trend was observed for the *a*- and *c*-parameters of the (Zr, Nb)_2_(Al, Sn)C double solid solution. A nearly single-phase 211 MAX material was obtained with a Zr:Nb ratio of 80:20 and Al:Sn ratio of 50:50, for which the rock-salt-like (Zr, Nb)C phase was eliminated. Moreover, an M:A ratio starting powder composition of 2:1.05 was used to minimize the amount of the ZrAl_2_ phase.

The effect of Nb and Sn on the crystallographic parameters was studied in detail. Neutron powder diffraction of Zr_2_SnC was performed to accurately determine the z-coordinate of Zr in the 211 unit cell. This analysis showed that the prismatic distortion in Zr_2_AlC is significantly larger than the octahedral distortion, and that the former can be reduced by the addition of Nb and Sn. The addition of Sn increased the octahedral distortion.

The analysis was expanded and the general crystallographic structure of the 211 MAX phase carbides was discussed. No phase stability range could be determined in terms of distortion parameters, but a correlation between the atomic radii of the M and A elements was proposed. The large M-atoms combine notably better with large A-atoms and the small M-atoms with small A-atoms. This match in atomic radii between M and A can be used as a practical guideline for the synthesis of other MAX phase solid solutions. In general, the concept of a double solid solution was found advantageous in terms of MAX phase synthesis and MAX phase purity.

## Electronic supplementary material

Supporting Information
