# Supplementary material for: The double solid solution (Zr, Nb)2(Al, Sn)C MAX phase: a steric stability approach
Source: Sci Rep. 2018 Aug 24;8:12801. doi: 10.1038/s41598-018-31271-2 (PMC6109093; doi:10.1038/s41598-018-31271-2)
Supplement: Supplementary file 1 — Supporting Information [file 41598_2018_31271_MOESM1_ESM.docx]

**Supplementary Information for:**

**“The double solid solution (Zr,Nb)_2_(Al,Sn)C MAX phase: a steric stability approach”**

Thomas Lapauw^a,b,*^, Bensu Tunca^a,b^, Daniel Potashnikov^c^, Asaf Pesach^d^, Offir Ozeri^e^, Jozef Vleugels^a^, and Konstantina Lambrinou^b^

^a^ KU Leuven, Department of Materials Engineering, Kasteelpark Arenberg 44, B-3001 Leuven, Belgium

^b^ SCK•CEN, Boeretang 200, B-2400 Mol, Belgium

^c^ Israel Atomic Energy Commission, P.O. Box 7061, Tel-Aviv 61070, Israel

^d^ Physics Department, Nuclear Research Centre – Negev, Beer-Sheva 84190, Israel

^e^ Reactor Department, Nuclear Research Center-Soreq, Yavne 81800, Israel

* Corresponding author. Address: KU Leuven, Department of Materials Engineering, Kasteelpark Arenberg 44, B-3001 Leuven, Belgium. Tel.: + 32 16 37 36 05; fax: + 32 16 32 19 90

E-mail address: thomas.lapauw@kuleuven.be (T. Lapauw)

Table S1. Quantified phase content (in wt%) of the different (Zr,Nb)_2_(Al,Sn)C ceramics by Rietveld refinement. The given Zr:Nb and Al:Sn ratios correspond to the composition of the starting powder. Labels ‘211’, ‘312’ and ‘413’ refer to the different MAX phase structures. The estimated error is in the range of 1 wt%.

| **Zr** | **Nb** | **Al** | **Sn** | **211** | **312** | **413** | **MC** | **Zr_2_Al_3_** | **(Zr,Nb)Al_2_** | **(Nb,Zr)Al_3_** |
| --- | --- | --- | --- | --- | --- | --- | --- | --- | --- | --- |
| 0 | 100 | 100 | 0 | 94 |  | 2 |  |  |  | 4 |
| 20 | 80 | 100 | 0 | 77 |  | 21 |  |  |  | 3 |
| 33 | 67 | 100 | 0 | 64 |  | 28 | 5 |  |  | 3 |
| 50 | 50 | 100 | 0 | 73 |  |  | 27 |  |  |  |
| 66 | 34 | 100 | 0 | 71 |  |  | 25 |  |  | 4 |
| 75 | 25 | 100 | 0 | 64 |  |  | 30 |  | 6 |  |
| 80 | 20 | 100 | 0 | 51 |  |  | 39 |  | 10 |  |
| 90 | 10 | 100 | 0 | 46 | 4 |  | 41 |  | 9 |  |
| 95 | 5 | 100 | 0 | 29 | 9 |  | 42 | 12 | 8 |  |
| 100 | 0 | 100 | 0 | 20 | 3 |  | 47 | 30 |  |  |
| 100 | 0 | 0 | 100 | 98 |  |  | 2 |  |  |  |
| 100 | 0 | 20 | 80 | 100 |  |  | 0 |  |  |  |
| 100 | 0 | 40 | 60 | 88 |  |  | 5 | 7 |  |  |
| 100 | 0 | 50 | 50 | 82 |  |  | 11 |  | 7 |  |
| 100 | 0 | 60 | 40 | 78 |  |  | 13 |  | 9 |  |
| 100 | 0 | 75 | 25 | 60 |  |  | 27 | 13 |  |  |
| 100 | 0 | 80 | 20 | 55 |  |  | 31 | 14 |  |  |
| 100 | 0 | 90 | 10 | 44 | 5 |  | 35 | 16 |  |  |
| 100 | 0 | 100 | 0 | 20 | 3 |  | 47 | 30 |  |  |
| 90 | 10 | 40 | 60 | 100 |  |  |  |  |  |  |
| 80 | 20 | 50 | 50 | 98 |  |  |  |  |  | 2 |
| 90 | 10 | 50 | 50 | 92 |  |  | 5 |  |  | 3 |
| 80 | 20 | 60 | 40 | 85 |  |  | 12 |  | 3 |  |
| 90 | 10 | 60 | 40 | 82 |  |  | 13 |  | 5 |  |
| 80 | 20 | 75 | 25 | 76 |  |  | 20 |  | 4 |  |
| 85 | 15 | 85 | 15 | 78 |  |  | 19 | 3 | 0 |  |
| 90 | 10 | 90 | 10 | 69 |  |  | 26 | 5 |  |  |


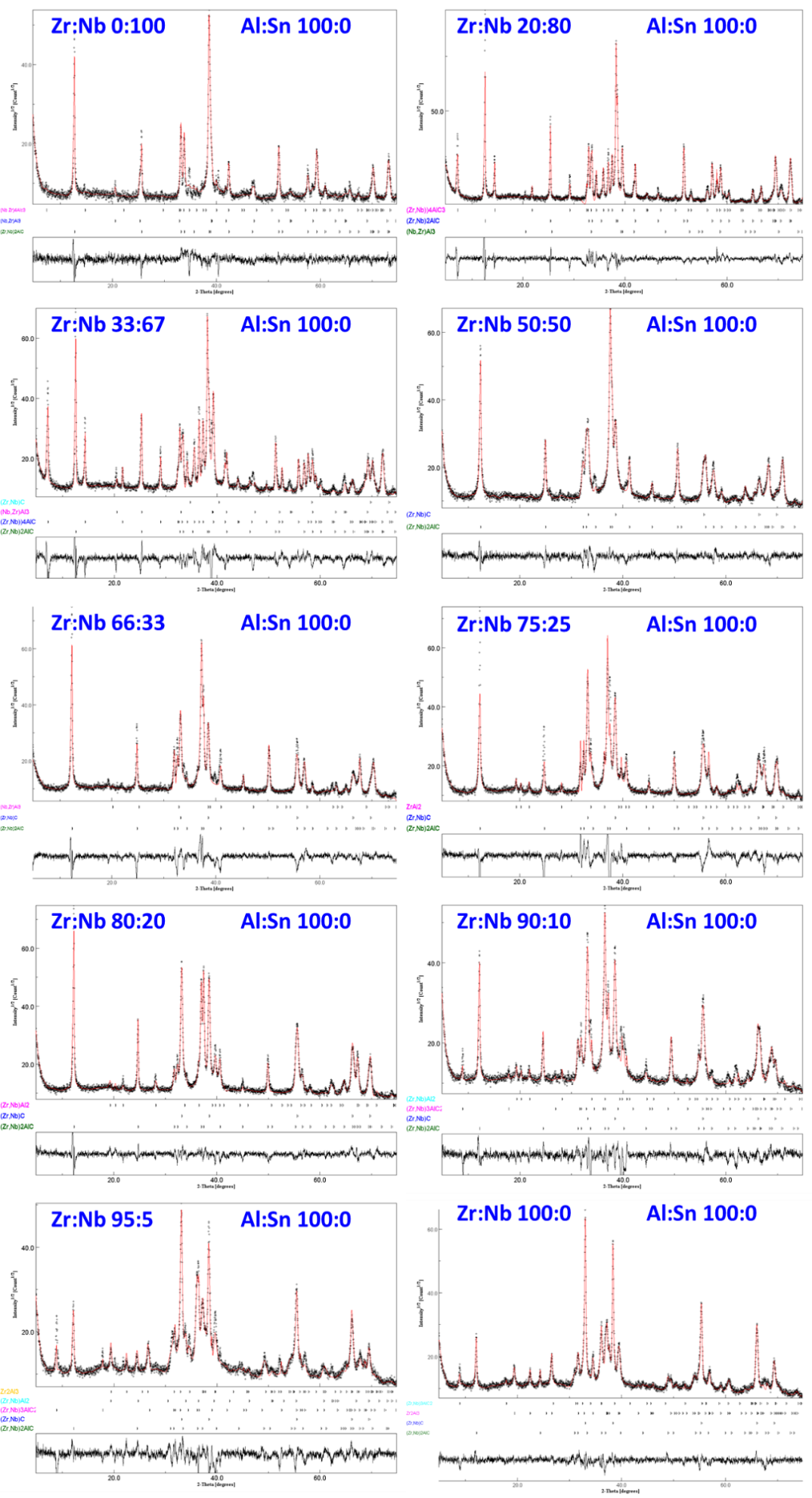


Figure S1. Rietveld refinement for the (Zr,Nb)_2_AlC ceramics. The black symbols indicate the XRD measurements, the red lines correspond to the calculated patterns. The difference between the observed and refined profile (black line) is shown in the lower part of each pattern.


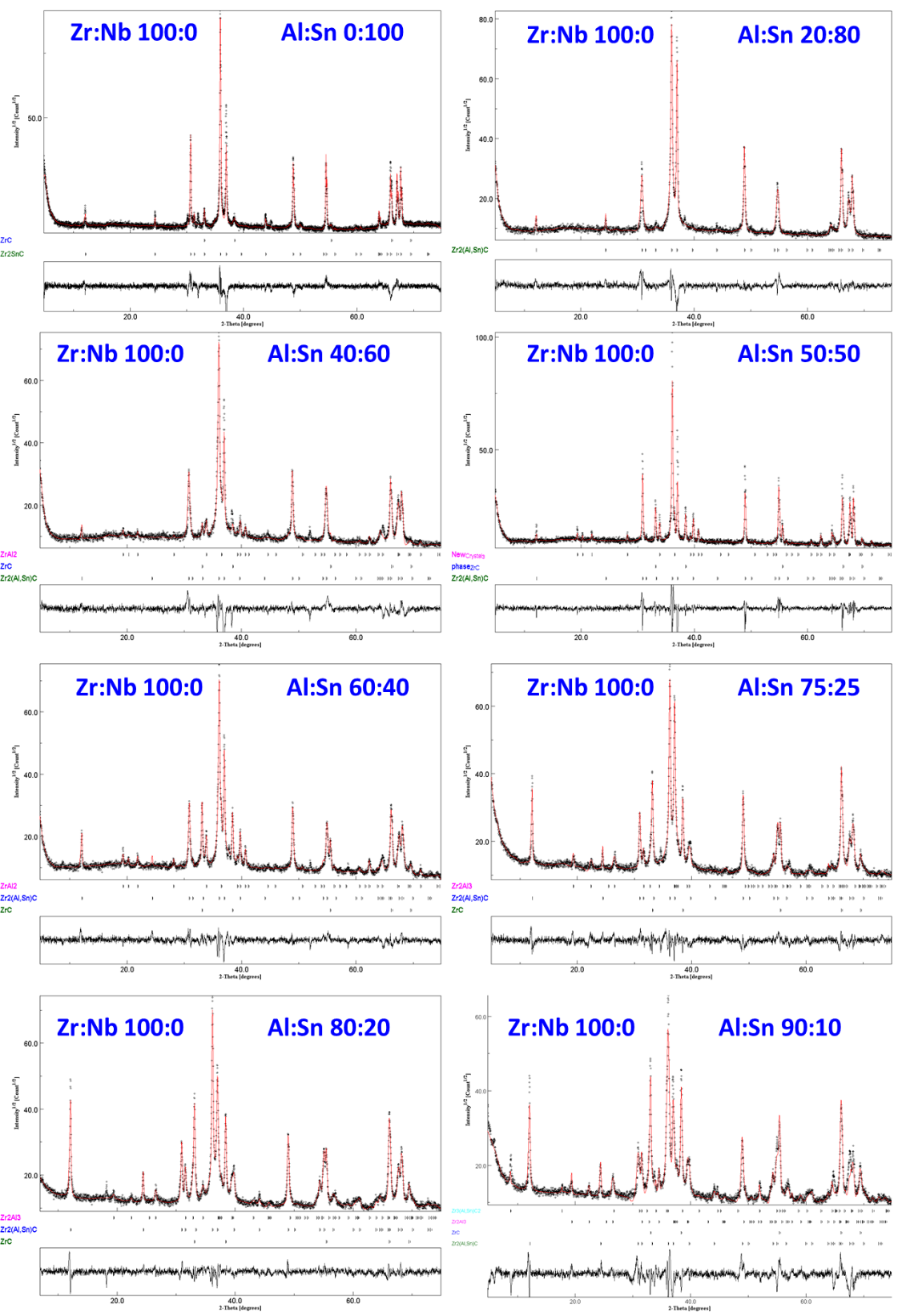


Figure S2. Rietveld refinement for the Zr_2_(Al,Sn)C ceramics. The black symbols indicate the XRD measurements, the red lines correspond to the calculated patterns. The difference between the observed and refined profile (black line) is shown in the lower part of each pattern.


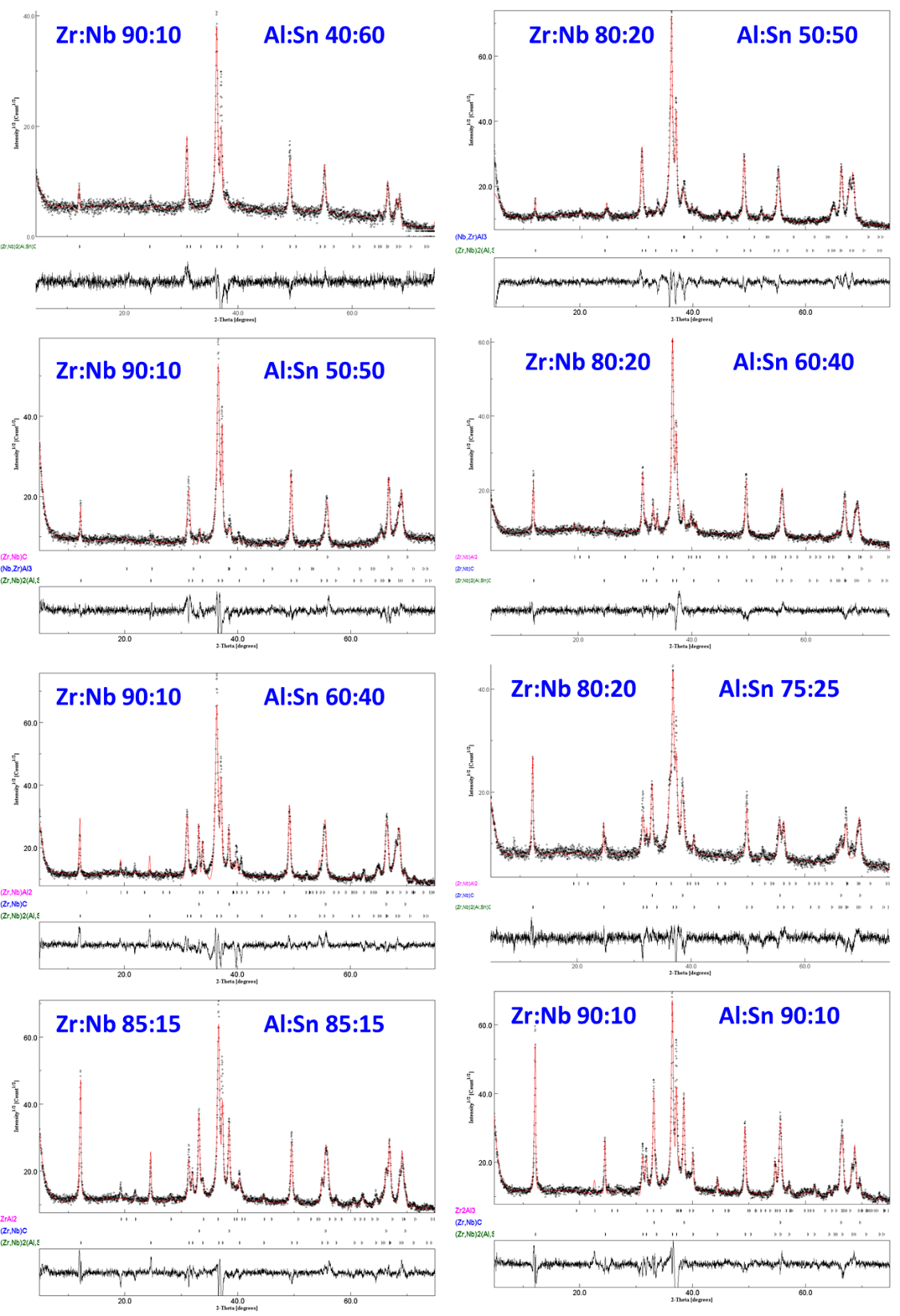


Figure S3. Rietveld refinement for the (Zr,Nb)_2_(Al,Sn)C ceramics. The black symbols indicate the XRD measurements, the red lines correspond to the calculated patterns. The difference between the observed and refined profile (black line) is shown in the lower part of each pattern.


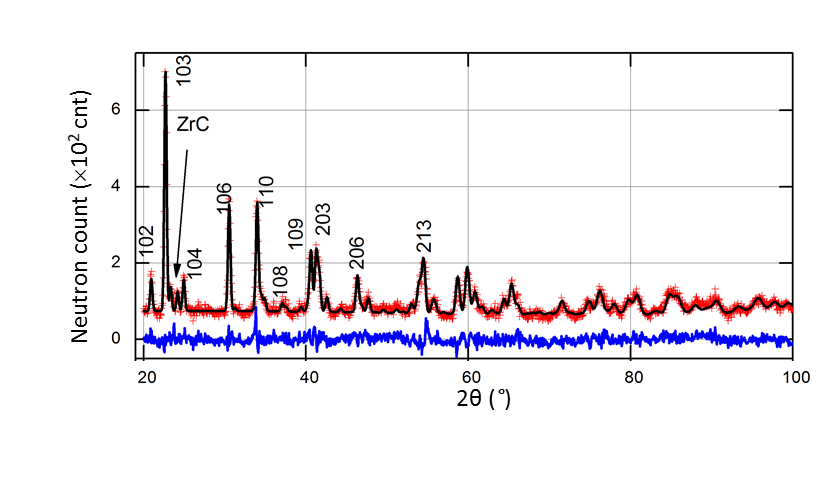


Figure S4. The measured neutron count (red symbols) of Zr_2_SnC and the respective Rietveld refined profile (black line) as function of the scattering angle, 2*θ*. The difference between the observed and refined profile (blue line) is shown in the lower part of the pattern. Some Zr_2_SnC reflections are indicated using the Miller indices together with one reflection which originate from ZrC impurities.
